# A comparison of 2D and 3D magnetic resonance imaging-based intratumoral and peritumoral radiomics models for the prognostic prediction of endometrial cancer: a pilot study

**DOI:** 10.1186/s40644-024-00743-2

**Published:** 2024-07-31

**Authors:** Ruixin Yan, Siyuan Qin, Jiajia Xu, Weili Zhao, Peijin Xin, Xiaoying Xing, Ning Lang

**Affiliations:** Department of Radiology, Peking University Third Hospital, 49 North Garden Road, Haidian District, Beijing, 100191 People’s Republic of China

**Keywords:** Magnetic resonance imaging, Endometrial cancer, Radiomics, Prognostic analysis

## Abstract

**Background:**

Accurate prognostic assessment is vital for the personalized treatment of endometrial cancer (EC). Although radiomics models have demonstrated prognostic potential in EC, the impact of region of interest (ROI) delineation strategies and the clinical significance of peritumoral features remain uncertain. Our study thereby aimed to explore the predictive performance of varying radiomics models for the prediction of LVSI, DMI, and disease stage in EC.

**Methods:**

Patients with 174 histopathology-confirmed EC were retrospectively reviewed. ROIs were manually delineated using the 2D and 3D approach on T2-weighted MRI images. Six radiomics models involving intratumoral (2D_intra_ and 3D_intra_), peritumoral (2D_peri_ and 3D_peri_), and combined models (2D_intra + peri_ and 3D_intra + peri_) were developed. Models were constructed using the logistic regression method with five-fold cross-validation. Area under the receiver operating characteristic curve (AUC) was assessed, and was compared using the Delong’s test.

**Results:**

No significant differences in AUC were observed between the 2D_intra_ and 3D_intra_ models, or the 2D_peri_ and 3D_peri_ models in all prediction tasks (*P* > 0.05). Significant difference was observed between the 3D_intra_ and 3D_peri_ models for LVSI (0.738 vs. 0.805) and DMI prediction (0.719 vs. 0.804). The 3D_intra + peri_ models demonstrated significantly better predictive performance in all 3 prediction tasks compared to the 3D_intra_ model in both the training and validation cohorts (*P* < 0.05).

**Conclusions:**

Comparable predictive performance was observed between the 2D and 3D models. Combined models significantly improved predictive performance, especially with 3D delineation, suggesting that intra- and peritumoral features can provide complementary information for comprehensive prognostication of EC.

**Supplementary Information:**

The online version contains supplementary material available at 10.1186/s40644-024-00743-2.

## Background

Endometrial cancer (EC) is the most common gynecological malignancy in developed country and second most common in China. Its incidence and mortality rate were still rising in recent years [1, 2]. Approximately 3% of women will develop EC in their lifetime with a median age of 61 years at first diagnosis [3]. While EC has an encouraging overall 5-year survival rate of 81% [4], clinicians face significant challenges in treatment decisions of overtreatment for low-risk patients and undertreatment for high-risk patients. Accurate risk assessment rely heavily on important histopathological factors such as International Federation of Gynecology and Obstetrics (FIGO) stage, lymphovascular space invasion (LVSI), and depth of myometrial invasion (DMI) [5–8]. While precisely determining these prognostic metrics continues to be crucial for distant relapse assessment and of poor prognosis estimation. However, such prognostic biomarkers can only be confirmed by histopathology, and is the main challenge of current clinical practice.

Magnetic resonance imaging (MRI) is considered mainstay for the preoperative evaluation of EC, given its high soft tissue contrast resolution allowing for the delineation of tumor invasion [1, 9]. However, the subjective nature in visual interpretation remains a limitation of conventional MRI. As reported by Arnaiz et al. assessing its role in disease staging, accurate prediction was only achieved in 47.2% of cases [10]. Radiomics represent a novel technique enabling the extraction of large amounts of data from medical images in a high-throughput and quantitative manner [11]. Since its advent, it has demonstrated great potential as a diagnostic and prognostic tool for a wide range of cancers [12–15]. In the context of EC, radiomics have demonstrated the value in not only the characterization of tumors, but also the prediction of high-risk diseases and survival outcomes [16–20].

Region of interest (ROI) delineation strategies can influence feature extraction and hence, affect the downstream performance of a radiomics model. Two approaches currently exist — the two-dimensional (2D) approach, which involves the delineation of lesions from a single image layer at the largest cross-section, or simply the center slice, and the three-dimensional (3D) approach, which involves the use of all tumor-containing slices. While the latter approach has been widely advocated for its comprehensiveness in tumor characterization [17, 20, 21], the need for manual delineation renders it more time-consuming and labor-intensive. As of now, the optimal ROI delineation strategy for the radiomics profiling of EC remains unclear. Besides, the majority of previous MRI-based radiomics research has placed focused on the characterization of intratumoral regions [16, 20, 21]. However, the surrounding microenvironment has been increasingly shown to offer insight into the clinical behavior of primary lesions [22–24].

This study thereby aimed to compare the clinical value of 2D and 3D MRI-based radiomics features for the prediction of LVSI, DMI, and disease stage in EC, as well as contribute to the existing literature on the significance of peritumoral radiomics features for prognostication of the disease.

## Methods

### Patient selection

Patients diagnosed with endometrial cancer between January 2017 and December 2022 at Peking University Third Hospital were retrospectively reviewed. All patients who underwent pelvic magnetic resonance imaging (MRI) within 14 days prior to hysterectomy were considered. The exclusion criteria included the following: (1) tumors with a maximum diameter of < 1 cm, (2) inadequate or poor image quality, (3) absence of relevant clinical or pathological information, (4) prior treatment with adjuvant chemotherapy or radiotherapy, and (5) non-endometrial primary malignancies.

This study was approved by the Ethics Committee of the institute (M2023637), who waived the requirement for informed patient consent.

### Clinicopathological data collection

Clinicopathological characteristics including age, DMI, LVSI, tumor stage, histopathological type and serum CA125 and CA199 levels were obtained from medical records. Histopathological assessment was performed on all surgical specimens. Tumor staging was performed according to the 2009 FIGO staging system, with stages I – II and III – IV were classified as early- and late-stage EC, respectively [25].

### Image acquisition and segmentation

Preoperative pelvic MRI was performed using 1.5 T Avanto scanner and 3.0 T Skyra scanners, with protocol parameters detailed in Supplementary Table S1. Axial T2-weighted images were acquired and used for subsequent analysis due to their ability to effectively distinguish between tumor-tissue and normal surrounding myometrial-tissue [1]. All images were imported into uAI Research Portal (V1.1, United Imaging Intelligence, Co., Ltd., Shanghai, China).

Intratumoral ROIs (ROI_intra_) were manually delineated by a radiologist (R.Y.) with 5-year experience, which were then reviewed and refined where necessary by a second radiologist (X.X.) with 15-year experience. Both radiologists were blinded to the study. ROI_intra_ was defined as the entire tumor volume across all tumor slices on 3D images, and as the maximum tumor cross-section on 2D images. Any cystic or hemorrhagic areas were avoided. Peritumoral ROIs (ROI_peri_) were automatically delineated by extending the tumor margin by 3 mm generated using the boundary “dilation” tool in the uAI platform [19, 24].

### Image preprocessing and radiomics feature extraction

Image preprocessing and radiomics feature extraction were performed in accordance to the Image Biomarker Standardisation Initiative (IBSI [26]). All T2-weighted images were resampled to an isotropic pixel size of 1 × 1 × 1 mm using BSpline interpolation to eliminate resolution discrepancies between devices. Pixel intensities were subsequently standardized to a common scale to further minimize background noise.

Radiomics features were extracted using the PyRadiomics python package. A total of 1197 radiomics features were extracted for each ROI, and consisted of the following 7 classes: first-order statistics (19.5%), shape-based features (1.2%), texture-based features including gray level co-occurrence matrix (GLCM) (23.9%) capturing texture, gray level run length matrix (GLRLM) (17.4%) describing gray level runs, gray level size zone matrix (GLSZM) (17.4%) characterizing zone sizes, neighboring gray tone difference matrix (NGTDM) (5.4%) quantifying differences between tumor and neighborhood pixels, and gray level dependence matrix (GLDM) (15.2%) analyzing dependency of gray levels. Subsequently Combined feature (ROI_intra+peri_) was fused by merging the extracted features from intra-tumoral models and respective peritumoral models. All extracted features were standardized using the Z-score normalization method to standardize the different measurement units across features, preventing model results from being influenced by feature-scale variations.

### Radiomic feature selection and machine learning model construction

First, features with correlation coefficients *P*-value less than 0.05 were remained. Least absolute shrinkage and selection operator regression (LASSO) was subsequently performed to select for those with non-zero coefficients. Five-fold cross-validation was applied, features appeared in more than two times were finally selected by “vote” function on the uAI platform to establish the radiomic models.

**Fig. 1:**
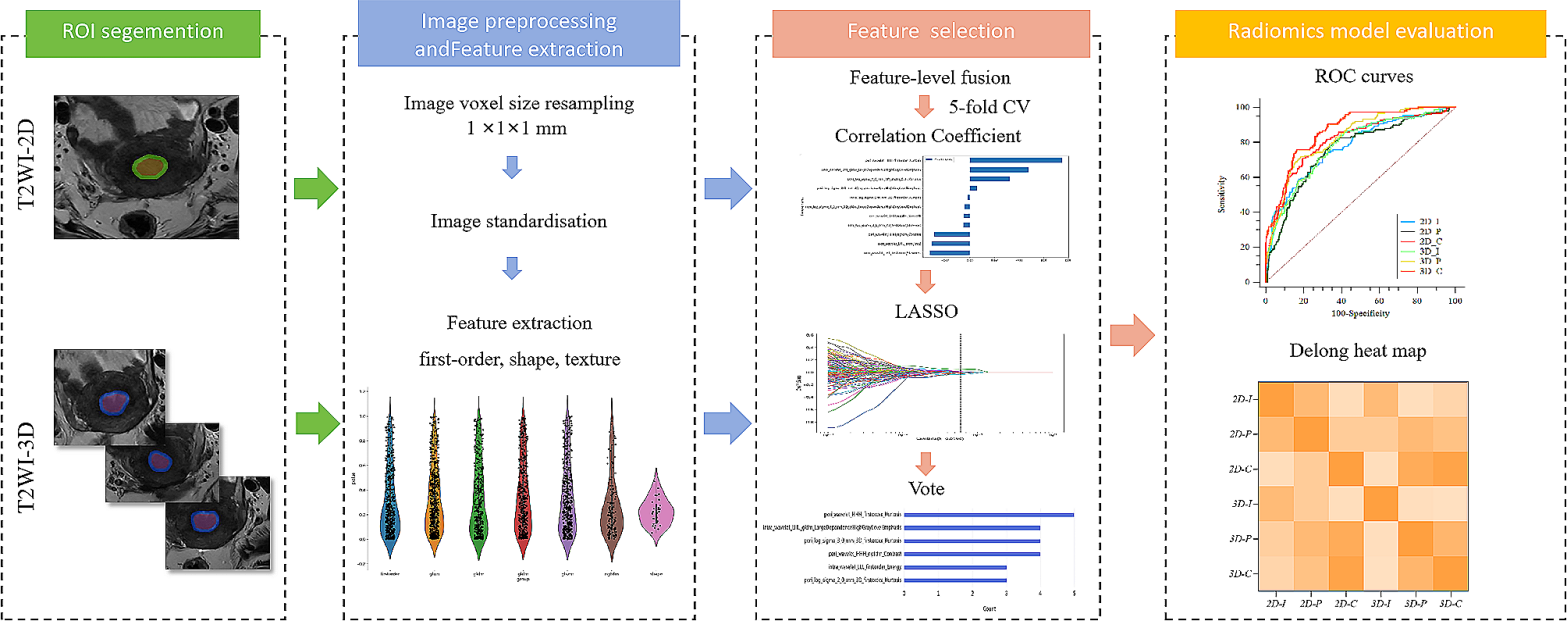
The radiomics workflow

The models were eventually constructed using selected radiomics features using logistic regression. Patients were randomly divided into five equal size subsets, one subset served as the validation set. The remaining four-fifths were the training set. This process was then repeated five times with different subsets for validation and training. The performance was averaged across the five times process to obtain a robust estimate of generalizability. The radiomics workflow is illustrated in Fig. 1.

### Statistical analysis

All statistical analyses were conducted using the SPSS software (IBM, Chicago, IL, USA, R23.0.0.0). Diagnostic performance was assessed using the receiver operating characteristic (ROC) curve analysis in terms of areas under the ROC curve (AUC), sensitivity, and specificity and accuracy. AUCs were compared using the Delong test. and are illustrated in the form of heatmaps.

## Results

### Baseline clinicopathological characteristics

**Fig. 2:**
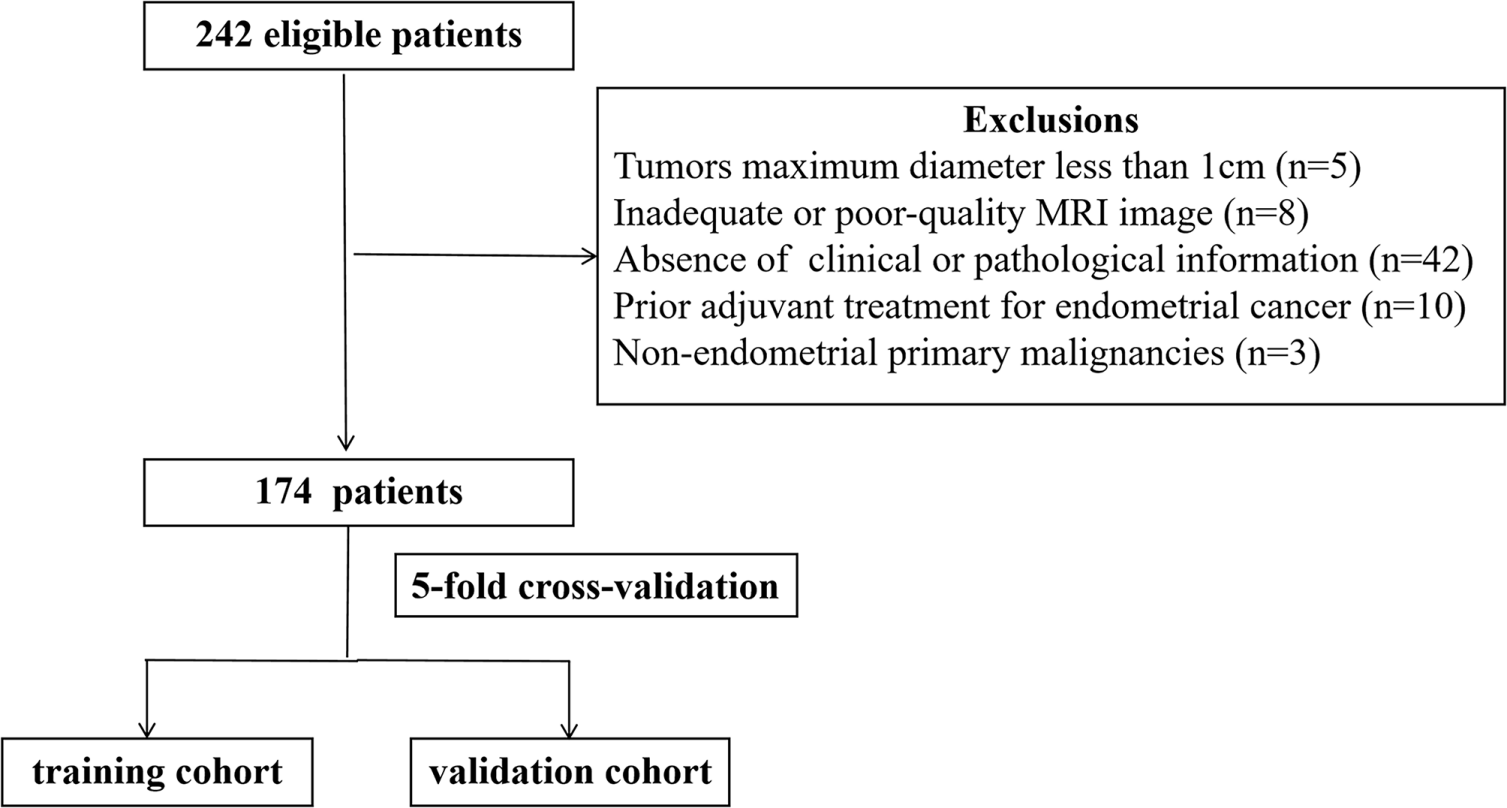
The patient selection process

A total of 174 patients with EC were enrolled in our study. The patient selection process is illustrated in Fig. 2. The average age was 56.96 ± 11.43 years (range, 27–86 years). Among them, 37 (21.3%) presented with late-stage disease. LVSI was observed in 39 (22.4%) patients, while DMI was seen in 55 (31.6%). The most common EC cell type was observed to be endometrioid adenocarcinoma (88.5%). All baseline characteristics of the patients are shown in Table 1.

**Table 1:** Baseline clinicopathological characteristics of the included patients

Variables	
Age (year), ± SD	56.96 ± 11.43
FIGO stage, n (%)	
Early-stage	137 (78.7%)
Late-stage	37 (21.3%)
LVSI, n (%)	
+	39 (22.4%)
-	135 (77.6%)
DMI, n (%)	
+	55 (31.6%)
-	119 (68.4%)
CA125 (u/ml), ± SD	34.97 ± 45.70
CA199 (u/ml), ± SD	40.60 ± 88.96
Type, n (%)	
Endometrioid carcinoma	154 (88.5%)
Non-Endometrioid carcinoma	20 (11.5%)

Abbreviations: FIGO stage, International Federation of Gynecology and Obstetrics (2009) stage, stages I– II and III – IV defined as early- and late-stage EC, respectively; LVSI, lymphovascular space invasion; and DMI, deep myometrial invasion; Non-Endometrioid carcinoma: include Serous carcinoma, Clear cell carcinoma, Mixed carcinoma, Undifferentiated carcinoma and Dedifferentiated carcinoma

### Radiomics feature selection

**Fig. 3:**
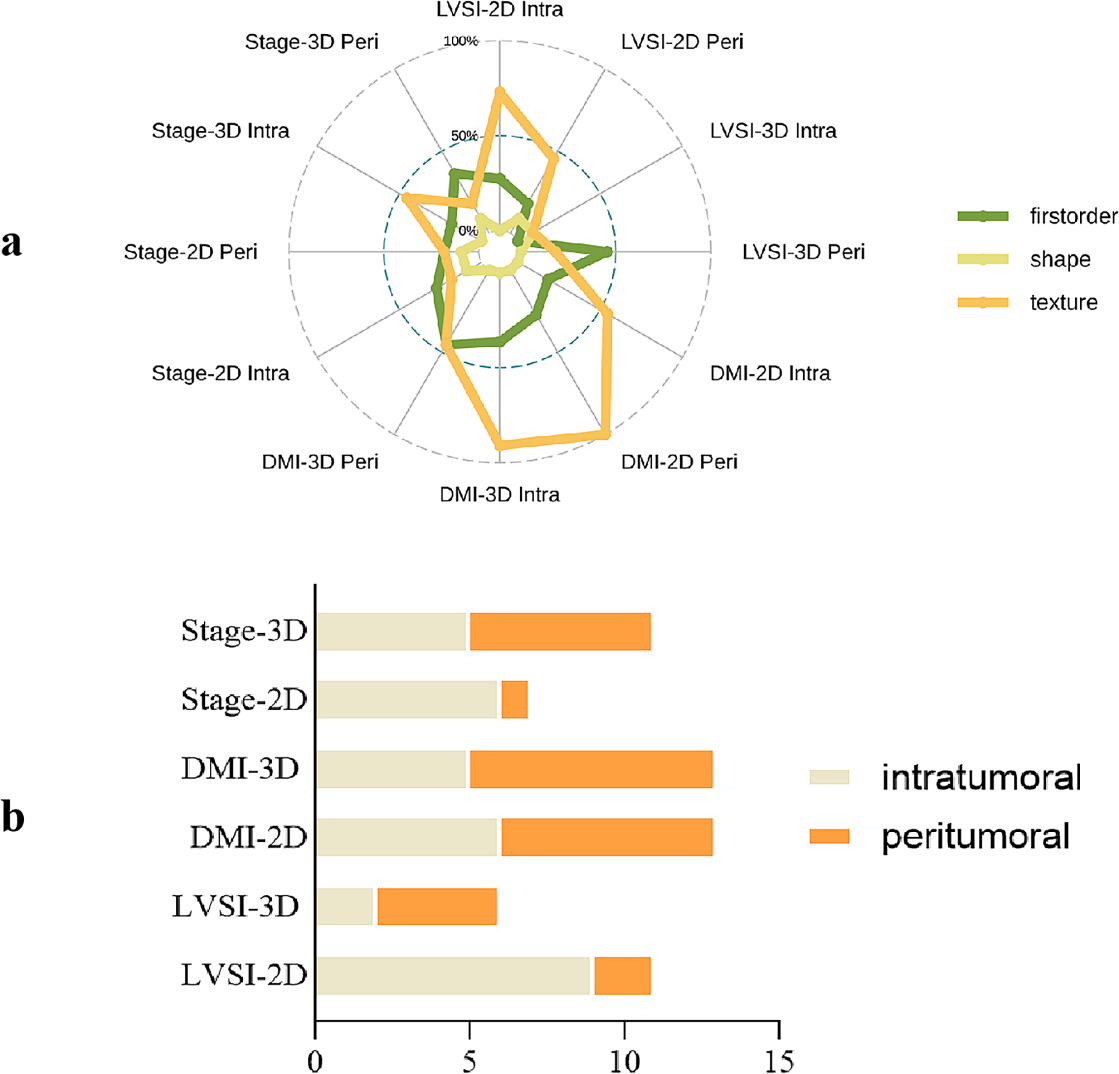
Distribution and composition of (**a**) the three feature classes (first-order, shape, texture) across the models, and (**b**) the intra- and peritumoral features across the models

Six models, 2D_intra_, 2D_peri_, 2D_intra + peri_, 3D_intra_, 3D_peri_, and 3D_intra + peri_, were constructed for each task. The total number of radiomics features retained in each model were as follows: 11, 8, 11, 2, 7, and 6, respectively, for LVSI prediction; 8, 14, 13, 14, 10, and 13, respectively, for DMI prediction; and 6, 6, 7, 7, 7, and 11, respectively, for FIGO stage prediction. The distribution of radiomics feature classes of intra- and peri-tumoral region across all models is shown in Fig. 3a. The number of intra- and peritumoral features incorporated into each model is shown in Fig. 3b. The exact feature names and type are provided in Supplementary Tables S2 – S4.

### Predictive performance of the independent radiomics models

**Fig. 4:**
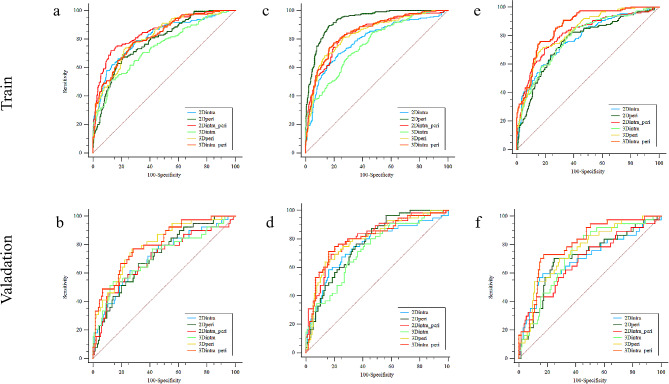
The receiver operating characteristic curve analysis results for (**a, b**) LVSI prediction, (**c, d**) DMI prediction, and (**e, f**) disease stage prediction

**Fig. 5:**
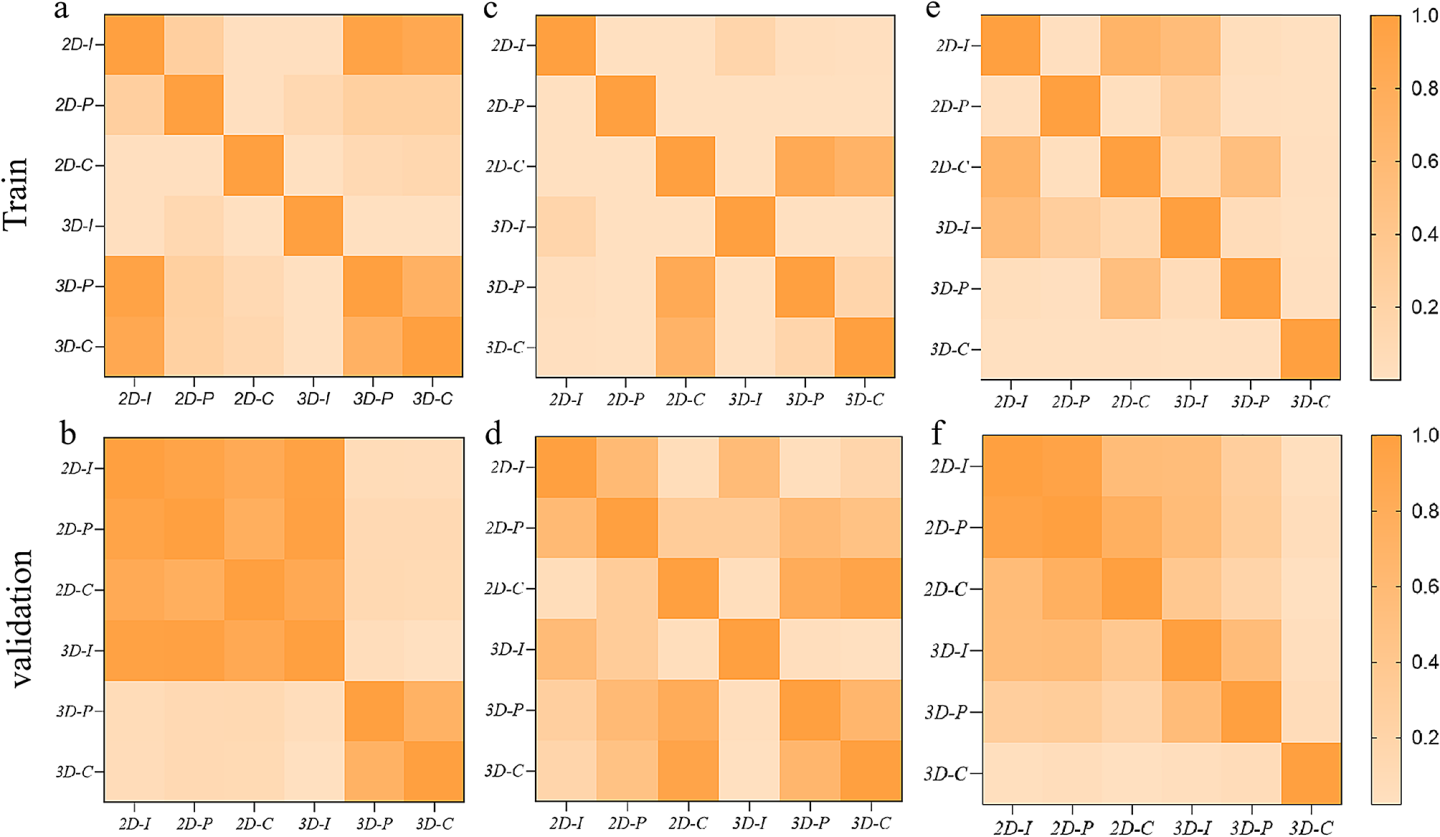
The Delong test results for (**a, b**) LVSI prediction, (**c, d**) DMI prediction, and (**e, f**) disease stage prediction between the training and validation cohorts, respectively

The ROC curve results of the six established models are shown in both Table 2; Fig. 4. The Delong test results are shown in Fig. 5 and Supplementary Tables S5 – S10.

**Table 2:** Logistic regression with five-fold cross-validation for predictive performance of the models

Model		Group	Mean AUC (95%CI)	ACC	SEN	SPE
LVSI	2D_intra_	Train	0.810 (0.718–0.903)	0.730	0.737	0.728
		Val	0.725 (0.505–0.937)	0.666	0.639	0.674
	2D_peri_	Train	0.784 (0.695–0.874)	0.682	0.731	0.669
		Val	0.706 (0.490–0.920)	0.644	0.668	0.637
	2D_intra + peri_	Train	0.853 (0.773–0.933)	0.730	0.789	0.713
		Val	0.720 (0.493–0.944)	0.667	0.639	0.674
	3D_intra_	Train	0.738 (0.634–0.844)	0.675	0.647	0.683
		Val	0.738 (0.534–0.933)	0.684	0.614	0.704
	3D_peri_	Train	0.813 (0.782–0.898)	0.726	0.769	0.713
		Val	0.805 (0.625–0.977	0.695	0.771	0.674
	3D_intra + peri_	Train	0.816 (0.730–0.901)	0.737	0.750	0.733
		Val	0.812 (0.633–0.979)	0.737	0.743	0.726
DMI	2D_intra_	Train	0.784 (0.699–0.871)	0.762	0.636	0.819
		Val	0.760 (0.582–0.938)	0.736	0.600	0.800
	2D_peri_	Train	0.928 (0.889–0.972)	0.826	0.936	0.775
		Val	0.773 (0.614–0.932)	0.684	0.727	0.663
	2D_intra + peri_	Train	0.839 (0.768–0.913)	0.783	0.741	0.803
		Val	0.799 (0.638–0.963)	0.753	0.691	0.782
	3D_intra_	Train	0.761 (0.679–0.847)	0.700	0.641	0.727
		Val	0.719 (0.538–0.904)	0.679	0.618	0.707
	3D_peri_	Train	0.833 (0.761–0.909)	0.757	0.791	0.742
		Val	0.804 (0.654–0.953)	0.724	0.745	0.715
	3D_intra + peri_	Train	0.845 (0.776–0.917)	0.779	0.782	0.777
		Val	0.807 (0.653–0.959)	0.770	0.745	0.782
Stage	2D_intra_	Train	0.773 (0.673–0.872)	0.703	0.703	0.703
		Val	0.704 (0.478–0.928)	0.625	0.701	0.683
	2D_peri_	Train	0.760 (0.658–0.860)	0.716	0.699	0.703
		Val	0.706 (0.491–0.919)	0.704	0.701	0.701
	2D_intra + peri_	Train	0.814 (0.719–0.909)	0.757	0.725	0.731
		Val	0.687 (0.472–0.896)	0.575	0.693	0.667
	3D_intra_	Train	0.782 (0.688–0.877)	0.663	0.732	0.717
		Val	0.738 (0.540–0.932)	0.621	0.694	0.678
	3D_peri_	Train	0.824 (0.747–0.908)	0.730	0.748	0.744
		Val	0.763 (0.581–0.942)	0.704	0.715	0.713
	3D_intra + peri_	Train	0.864 (0.795–0.933)	0.717	0.854	0.825
		Val	0.804 (0.640–0.967)	0.700	0.833	0.805

Abbreviations: AUC, area under the receiver operating characteristic curve; ACC, accuracy; SEN: sensitivity, and SPE: specificity

In terms of ROI delineation strategies, significant differences were observed between the 2D_intra_ and 3D_intra_ models for LVSI prediction in the training cohort [0.810 (0.718–0.903) vs. 0.738 (0.634–0.844), *P* = 0.001]. Additionally, significant differences were shown between the 2D_peri_ and 3D_peri_ models for both DMI [0.928 (0.889–0.972) vs. 0.833 (0.761–0.909), *P* = 0.000] and stage prediction [0.760 (0.658–0.860) vs. 0.824 (0.747–0.908), *P* = 0.000] in the training data. Such significant differences were not observed between all models in the validation cohort (*P* > 0.05).

In terms of intra-and peri-tumoral features, significant differences were observed between the 2D_intra_ and 2D_peri_ models for DMI prediction [0.784 (0.699–0.871) vs. 0.928 (0.889–0.972, *P* = 0.000] in the training cohort, which was not observed in the validation cohort (*P* > 0.05). Significant differences were observed between the 3D_intra_ versus 3D_peri_ models for LVSI [0.738 (0.634–0.844) vs. 0.813 (0.782–0.898), *P* = 0.000], DMI [0.761 (0.679–0.847) vs. 0.833 (0.761–0.909), *P* = 0.000], and stage prediction [0.782 (0.688–0.877) vs. 0.824 (0.747–0.908), *P* = 0.049)] in the training cohort. Such significance remained in the validation cohort for LVSI prediction [0.738 (0.634–0.844) vs. 0.805 (0.625–0.977), *P* = 0.049] and DMI prediction [0.719 (0.538–0.904) vs. 0.804 (0.654–0.953), *P* = 0.019], but not for stage prediction [0.738 (0.540–0.932) vs. 0.763 (0.581–0.942].

### Predictive performance of the combined radiomics models

For LVSI prediction, the 2D_intra + peri_ and 3D_intra + peri_ models demonstrated AUC values of 0.853 (0.773–0.933) and 0.816 (0.730–0.901), respectively, in the training cohort and values of 0.720 (0.493–0.944) and 0.812 (0.633–0.979), respectively, in the validation cohort. For DMI prediction, the AUC values were 0.839 (0.768–0.913) and 0.845 (0.776–0.917), respectively, in the training cohort, and were 0.799 (0.638–0.963) and 0.807 (0.653–0.959), respectively, in the validation cohort. For stage prediction, significant differences were demonstrated in both the training cohort [0.814 (0.719–0.909) vs. [0.864 (0.795–0.933), *P* = 0.006] and the validation cohort [0.687 (95% CI, 0.472–0.896) vs. 0.804 (0.640–0.967), *P* = 0.023] in the validation cohort.

Compared to the 2D_intra_ model, significantly increase in predictive performance was observed with inclusion of peritumoral features for LVSI (+ 0.043, *P* = 0.006) and DMI (+ 0.055, (*P* = 0.000)) in the training cohort. Significant increase was observed for DMI prediction (+ 0.039, *P* = 0.045) in the validation cohort. Compared to the 3D_intra_ model, significant increase in prediction efficacy was observed for LVSI prediction (+ 0.078, *P* = 0.000), DMI prediction (+ 0.084, *P* = 0.000), and FIGO stage prediction (+ 0.066, *P* = 0.000) in the training cohort. Such significance was also observed for LVSI prediction (+ 0.074, *P* = 0.018), DMI prediction (+ 0.088, *P* = 0.003), and FIGO stage prediction (+ 0.066, *P* = 0.039) in the validation cohort.

## Discussion

Six prognostic radiomics models of various ROI delineation strategies were constructed in this study, all of which yielded favorable diagnostic performance, demonstrating the potential as non-invasive tools for the prediction of LVSI, DMI, and FIGO stage to guide the management of EC. Our study further found comparable results between the 2D and 3D radiomics models, implying the lack of added value with the more labor-intensive 3D delineation approach. Our findings also demonstrated that assessment of combined intra- and peritumoral features can further enhance the predictive ability of the model.

There has been ongoing debate regarding the use of 2D and 3D radiomics features. Several studies have reported the superiority of 3D models in capturing tumor characteristics, Ortiz-Ramón et al. employed 2D and 3D MRI radiomics to differentiate the origins of brain metastases. Machado et al. utilized MRI radiomics to predict recurrence in clinically non-functioning pituitary macroadenomas. Watzenboeck et al. employed MRI radiomics to assess fetal lung development [27–29], but are often limited in sample size and in their overlooking of peritumoral regions for feature extraction. Our study found comparable performance between 2D and 3D approaches both for intra- and peritumoral ROIs, which is in line with the those of the study by Zhang et al. for tumor phenotype prediction in non-small cell lung carcinomas [30], and the study by Arefan et al. for axillary lymph node metastasis risk in breast cancer [31], which also reported similar performance between 2D and 3D radiomics approaches. However, it’s important to note that findings in EC have been mixed. Fasmer et al. [25], using a relatively small sample size, found that 3D whole-tumor radiomic signatures yielded comparable AUC in the training set but significantly lower AUC in the validation set for advanced FIGO stage prediction in EC. These varying results across studies highlight the need for further investigation into the optimal radiomics approach for EC, particularly with larger sample sizes and robust validation.

Such observations may be attributable to the inevitable amplification of noise as the whole tumor volume is delineated across multiple image slices. While 2D ROI delineation within a representative slicing plane may encapsulate key characteristics of the entire tumor volume. Our study highlighted that despite the potential for additional information provided in assessing the entire tumor volume, the marginal gains in predictive ability may not outweigh the practical demands associated with manual multi-slice annotation. This observation could provide support for the promising model performance reported in previous literature, where the utilization of a single slice yielded comparable results [28]. Nonetheless, large-sample studies are warranted for verification of our results.

The growing recognition of the role of the tumor microenvironment in cancer biology has resulted in the recent shift in focus from the primary tumor itself to the surrounding stroma for radiomics feature extraction [32, 33]. Such significance in prognostic value of surrounding tissues has been demonstrated in breast [34] and ovarian cancers [35]. In this study, we simultaneously developed predictive models through an automated 3 mm boundary expansion of the intratumoral ROI, aimed at capturing spatial characteristics beyond the primary tumor. To our knowledge, this is the first study to compare the effects of 2D versus 3D ROI delineation approaches on peritumoral region. Our results demonstrated useful information that combined models can indeed be attained by inclusion of peritumoral lesion, showed highest diagnostic performance in both the 2D and 3D models, especially 3D models. The obtained AUC results demonstrate reasonable performance and are consistent with those of the study by Yan et al., who demonstrated the complementary nature of intra- and peritumoral features for LVSI and DMI prediction with AUCs of 0.859 and 0.856 respectively [22, 23]. This indicates that, compared to intratumoral features alone, peritumoral regions provide supplementary prognostic information. When coupled with intratumoral radiomics, this minimally invasive multi-regional segmentation provided a balanced assessment of the local tumor-stroma interplay for improved prognostic modeling.

Interesting, the 3D_peri_ model was observed to significantly outperform the 3D_intra_ model in our study. This may suggest that tissues in direct contact with the tumor are likely to undergo microstructural alterations prior to histologic changes. 3D peritumoral radiomics may thus offer the sensitivity required for identifying such pre-invasive alterations, and enable a more comprehensive characterization of spatial heterogeneity in the tumor microenvironment. Based on our findings, we propose the potential clinical utility of sampling adjacent stromal tissues, in addition to the primary lesion, for the histopathological assessment of ECs.

Our study had several limitations. First, the retrospective design and relatively modest sample size may limit the generalizability of our findings. Larger prospective studies are thereby needed to validate our results. Second, only a narrow set of histopathological variables with unbalanced cohorts were included due to data availability constraints, these differences could influence the radiomics features extracted and potentially impact the performance of our models. While our study was predominantly endometrioid adenocarcinoma, the validation results demonstrated good stability across the iterations of cross-validation. This suggests that despite pathology variability not fully accounted for, the validation approach helped improve generalizability to these cases Third, the optimal extent of peritumoral expansion was not explored in this study. Fourth, manual delineation may have inevitably resulted in inter-observer variability, which could have been mitigated by utilization of a semi-automated or automated approach. Lastly, more advanced hyperparameter optimization and deep learning methods were not applied due to the sample size of this study.

## Conclusions

This study developed and investigated the role of 2D and 3D intratumoral and peritumoral radiomics models in the prediction of LVSI, DMI, and disease stage in EC. A 3D ROI delineation approach did not achieve significant improvement in prediction performance. Inclusion of peritumoral features significantly enhanced prediction accuracy of the models, especially the 3D model. Further studies with standardized delineation strategies are warranted to validate our results and advance the clinical translation of radiomics in the field of gynecological oncology.

### Electronic supplementary material

Below is the link to the electronic supplementary material.

Supplementary Material 1

## Data Availability

The datasets used or analysed during the current study are available from the corresponding author on reasonable request.

## Abbreviations

EC: Endometrial cancer
FIGO: International Federation of Gynecology and Obstetrics
LVSI: Lymphovascular space invasion
DMI: Depth of myometrial invasion
MRI: Magnetic resonance imaging
3D: Three-dimensional
2D: Two-dimensional
GLCM: Gray level co-occurrence matrix
GLRLM: Gray level run length matrix
GLSZM: Gray level size zone matrix
NGTDM: Gray tone difference matrix
GLDM: Gray level dependence matrix
LASSO: Least absolute shrinkage and selection operator regression
ROC: Receiver operating characteristic
AUC: Areas under the ROC curve
2D_intra_: Two-dimensional intratumoral model
2D_peri_: Two-dimensional peritumoral model
2D_intra + peri_: Two-dimensional intratumoral + peritumoral model
3D_intra_: Three-dimensional intratumoral model
3D_peri_: Three-dimensional peritumoral model
3D_intra + peri_: Three-dimensional intratumoral + peritumoral model
