# Supplementary material for: A comparison of 2D and 3D magnetic resonance imaging-based intratumoral and peritumoral radiomics models for the prognostic prediction of endometrial cancer: a pilot study
Source: Cancer Imaging. 2024 Jul 31;24:100. doi: 10.1186/s40644-024-00743-2 (PMC11293005; doi:10.1186/s40644-024-00743-2)
Supplement: Supplementary file 1 — Supplementary Material 1 [file 40644_2024_743_MOESM1_ESM.docx]

| Table S1 Preoperative Female Pelvic T2WI Scanning Parameters | | | | |
| --- | --- | --- | --- | --- |
| Parameters | uMR Omega | GE 750W | SE Prisma | uMR 660i |
| Field Strength | 3T | 3T | 3T | 1.5T |
| TR/TE (ms) | 4230/127 | 5726/104.608 | 3830/86 | 5150/103.68 |
| FA (deg) | 135 | 111 | 159 | 150 |
| Slice/Gap (mm) | 4/0.8 | 4/0.4 | 4/0.4 | 4/0.4 |
| Matrix | 336x302 | 320x224 | 288×230 | 384×384 |
| FOV (mm) | 220x220 | 220x220 | 220x220 | 220x220 |
| Pixel(mm) | 0.73x0.65 | 0.98x0.69 | 0.60x0.60 | 0.90x0.81 |
| Averages | 1.5 | 2 | 1 | 2 |

TR: repetition time; TE:echo time; FA: flip angle; Gap: Gap between slices; FOV: field of view.

Table S2: Composition of selected radiomics features for LVSI task

| Model | No. | Features name |
| --- | --- | --- |
| 2D-I | 11 | \| intra_wavelet_HLL_glszm_LargeAreaHighGrayLevelEmphasis \| \| --- \| \| intra_log_sigma_2_0_mm_3D_gldm_LargeDependenceHighGrayLevelEmphasis \| \| intra_wavelet_LHH_gldm_LargeDependenceHighGrayLevelEmphasis \| \| intra_wavelet_HHL_gldm_LargeDependenceHighGrayLevelEmphasis \| \| intra_log_sigma_5_0_mm_3D_glszm_SmallAreaEmphasis \| \| intra_wavelet_HLH_glcm_InverseVariance \| \| intra_wavelet_HLH_firstorder_Kurtosis \| \| intra_wavelet_LLL_firstorder_TotalEnergy \| \| intra_wavelet_LLH_glcm_InverseVariance \| \| intra_wavelet_LHL_firstorder_Skewness \| \| intra_wavelet_LHL_gldm_LargeDependenceHighGrayLevelEmphasis \| |
| 2D-P | 8 | \| peri_original_shape_Maximum2DDiameterColumn \| \| --- \| \| peri_wavelet_LHH_glszm_SmallAreaEmphasis \| \| peri_wavelet_LLL_firstorder_Energy \| \| peri_wavelet_LLL_firstorder_TotalEnergy \| \| peri_log_sigma_5_0_mm_3D_gldm_SmallDependenceLowGrayLevelEmphasis \| \| peri_log_sigma_5_0_mm_3D_glcm_InverseVariance \| \| peri_log_sigma_5_0_mm_3D_glcm_Idn \| \| peri_wavelet_LLH_glszm_SizeZoneNonUniformityNormalized \| |
| 2D-C | 11 | \| intra_wavelet_HLL_glszm_LargeAreaHighGrayLevelEmphasis \| \| --- \| \| intra_wavelet_LLL_firstorder_TotalEnergy \| \| intra_wavelet_LHL_gldm_LargeDependenceHighGrayLevelEmphasis \| \| peri_wavelet_LHH_glszm_SmallAreaEmphasis \| \| intra_wavelet_LHH_gldm_LargeDependenceHighGrayLevelEmphasis \| \| intra_log_sigma_2_0_mm_3D_gldm_LargeDependenceHighGrayLevelEmphasis \| \| intra_wavelet_HHL_gldm_LargeDependenceHighGrayLevelEmphasis \| \| peri_log_sigma_5_0_mm_3D_glcm_Idn \| \| intra_wavelet_HLH_firstorder_Kurtosis \| \| intra_wavelet_HLH_glcm_InverseVariance \| \| intra_wavelet_LHL_firstorder_Skewness \| |
| 3D-I | 2 | \| intra_wavelet_LHL_gldm_LargeDependenceHighGrayLevelEmphasis \| \| --- \| \| intra_original_shape_LeastAxisLength \| |
| 3D-P | 7 | \| peri_wavelet_LLL_firstorder_Energy \| \| --- \| \| peri_wavelet_HHH_firstorder_Kurtosis \| \| peri_wavelet_LLL_firstorder_TotalEnergy \| \| peri_wavelet_HHH_ngtdm_Contrast \| \| peri_log_sigma_3_0_mm_3D_firstorder_Kurtosis \| \| peri_wavelet_HLH_glcm_Imc1 \| \| peri_log_sigma_2_0_mm_3D_firstorder_Kurtosis \| |
| 3D-C | 6 | \| peri_wavelet_HHH_firstorder_Kurtosis \| \| --- \| \| intra_wavelet_LHL_gldm_LargeDependenceHighGrayLevelEmphasis \| \| peri_log_sigma_3_0_mm_3D_firstorder_Kurtosis \| \| peri_wavelet_HHH_ngtdm_Contrast \| \| intra_wavelet_LLL_firstorder_Energy \| \| peri_log_sigma_2_0_mm_3D_firstorder_Kurtosis \| |

| Table S3: Composition of selected radiomics features for DMI task | | |
| --- | --- | --- |
| Model | No. | Features name |
| 2D-I | 8 | \| intra_log_sigma_5_0_mm_3D_firstorder_90Percentile \| \| --- \| \| intra_log_sigma_3_0_mm_3D_firstorder_Kurtosis \| \| intra_wavelet_LHH_gldm_LargeDependenceHighGrayLevelEmphasis \| \| intra_log_sigma_5_0_mm_3D_glszm_SmallAreaEmphasis \| \| intra_wavelet_HHL_glrlm_LongRunLowGrayLevelEmphasis \| \| intra_wavelet_LHL_glszm_SizeZoneNonUniformity \| \| intra_wavelet_HLH_glcm_Correlation \| \| intra_log_sigma_2_0_mm_3D_firstorder_Kurtosis \| |
| 2D-P | 14 | \| peri_wavelet_LLH_gldm_LargeDependenceLowGrayLevelEmphasis \| \| --- \| \| peri_log_sigma_5_0_mm_3D_glszm_SmallAreaEmphasis \| \| peri_log_sigma_4_0_mm_3D_glszm_SmallAreaEmphasis \| \| peri_wavelet_HLL_firstorder_Skewness \| \| peri_original_gldm_LargeDependenceLowGrayLevelEmphasis \| \| peri_log_sigma_5_0_mm_3D_glcm_InverseVariance \| \| peri_wavelet_LLH_gldm_LargeDependenceEmphasis \| \| peri_wavelet_LLH_firstorder_Median \| \| peri_wavelet_LLL_gldm_LargeDependenceLowGrayLevelEmphasis \| \| peri_log_sigma_4_0_mm_3D_glrlm_RunLengthNonUniformityNormalized \| \| peri_log_sigma_2_0_mm_3D_gldm_LargeDependenceHighGrayLevelEmphasis \| \| peri_log_sigma_2_0_mm_3D_firstorder_Skewness \| \| peri_wavelet_LLH_glrlm_ShortRunEmphasis \| \| peri_log_sigma_4_0_mm_3D_glszm_SizeZoneNonUniformity \| |
| 2D-C | 13 | \| intra_log_sigma_3_0_mm_3D_firstorder_Kurtosis \| \| --- \| \| intra_wavelet_LHH_gldm_LargeDependenceHighGrayLevelEmphasis \| \| peri_wavelet_HLL_firstorder_Skewness \| \| peri_wavelet_LLH_gldm_LargeDependenceLowGrayLevelEmphasis \| \| peri_wavelet_LLH_firstorder_Median \| \| intra_log_sigma_5_0_mm_3D_firstorder_90Percentile \| \| peri_wavelet_LLL_glszm_LargeAreaLowGrayLevelEmphasis \| \| peri_log_sigma_2_0_mm_3D_gldm_LargeDependenceHighGrayLevelEmphasis \| \| peri_wavelet_LLH_glrlm_ShortRunEmphasis \| \| intra_wavelet_HHL_glrlm_LongRunLowGrayLevelEmphasis \| \| intra_wavelet_LLH_firstorder_Kurtosis \| \| intra_wavelet_HLH_glcm_Correlation \| \| peri_original_gldm_LargeDependenceLowGrayLevelEmphasis \| |
| 3D-I | 14 | \| intra_log_sigma_5_0_mm_3D_glcm_Imc2 \| \| --- \| \| intra_log_sigma_5_0_mm_3D_firstorder_90Percentile \| \| intra_wavelet_LLH_firstorder_Median \| \| intra_wavelet_LLH_firstorder_Kurtosis \| \| intra_wavelet_LHL_glszm_SizeZoneNonUniformity \| \| intra_wavelet_LLH_glcm_MaximumProbability \| \| intra_log_sigma_5_0_mm_3D_glszm_LargeAreaLowGrayLevelEmphasis \| \| intra_original_glcm_Imc2 \| \| intra_wavelet_LLH_gldm_LargeDependenceHighGrayLevelEmphasis \| \| intra_log_sigma_3_0_mm_3D_gldm_LargeDependenceHighGrayLevelEmphasis \| \| intra_wavelet_HLL_glszm_LargeAreaLowGrayLevelEmphasis \| \| intra_wavelet_HLH_firstorder_10Percentile \| \| intra_log_sigma_5_0_mm_3D_ngtdm_Complexity \| \| intra_log_sigma_5_0_mm_3D_glszm_SizeZoneNonUniformityNormalized \| |
| 3D-P | 10 | \| peri_original_firstorder_Kurtosis \| \| --- \| \| peri_log_sigma_2_0_mm_3D_firstorder_Maximum \| \| peri_wavelet_LHH_firstorder_Minimum \| \| peri_wavelet_LHH_glcm_ClusterShade \| \| peri_wavelet_HHL_firstorder_Kurtosis \| \| peri_log_sigma_3_0_mm_3D_glszm_ZoneVariance \| \| peri_log_sigma_3_0_mm_3D_glszm_LargeAreaHighGrayLevelEmphasis \| \| peri_wavelet_HHH_firstorder_Kurtosis \| \| peri_log_sigma_4_0_mm_3D_glcm_ClusterShade \| \| peri_wavelet_HLH_glcm_ClusterShade \| |
| 3D-C | 13 | \| peri_wavelet_HHL_firstorder_Kurtosis \| \| --- \| \| intra_log_sigma_5_0_mm_3D_glcm_Imc2 \| \| intra_log_sigma_5_0_mm_3D_firstorder_90Percentile \| \| peri_original_firstorder_Kurtosis \| \| peri_log_sigma_2_0_mm_3D_firstorder_Maximum \| \| peri_wavelet_LHH_glcm_ClusterShade \| \| peri_log_sigma_5_0_mm_3D_glcm_ClusterShade \| \| peri_log_sigma_3_0_mm_3D_glszm_ZoneVariance \| \| intra_wavelet_LLH_firstorder_Median \| \| peri_wavelet_LHH_firstorder_Minimum \| \| intra_wavelet_HLL_ngtdm_Busyness \| \| intra_original_glszm_LargeAreaLowGrayLevelEmphasis \| \| peri_wavelet_HLH_glcm_ClusterShade \| |

| Table S4: Composition of selected radiomics features for Stage task | | |
| --- | --- | --- |
| Model | No. | Features name |
| Grade |  |  |
| 2D-I | 6 | \| intra_wavelet_LHL_firstorder_Kurtosis \| \| --- \| \| intra_wavelet_HLL_glszm_LargeAreaHighGrayLevelEmphasis \| \| intra_wavelet_LLL_firstorder_10Percentile \| \| intra_original_shape_Sphericity \| \| intra_wavelet_LLL_firstorder_Minimum \| \| intra_wavelet_LHH_glcm_ClusterShade \| |
| 2D-P | 6 | \| \| peri_original_shape_Maximum2DDiameterColumn \| \| --- \| \| peri_wavelet_LLL_firstorder_Skewness \| \| peri_log_sigma_2_0_mm_3D_ngtdm_Coarseness \| \| peri_wavelet_LLL_firstorder_Median \| \| peri_wavelet_LLH_glcm_JointEnergy \| \| peri_original_shape_Flatness \| \| \| --- \| --- \| --- \| --- \| --- \| --- \| --- \| |
| 2D-C | 7 | \| \| intra_wavelet_HLL_glszm_LargeAreaHighGrayLevelEmphasis \| \| --- \| \| intra_wavelet_LHL_firstorder_Kurtosis \| \| intra_wavelet_LLL_firstorder_10Percentile \| \| peri_wavelet_HHL_gldm_LargeDependenceLowGrayLevelEmphasis \| \| intra_wavelet_LLL_firstorder_Minimum \| \| intra_original_shape_Sphericity \| \| intra_wavelet_LHH_glcm_ClusterShade \| \| \| --- \| --- \| --- \| --- \| --- \| --- \| --- \| --- \| |
| 3D-I | 7 | \| \| intra_wavelet_LHH_glcm_ClusterShade \| \| --- \| \| intra_original_glrlm_ShortRunEmphasis \| \| intra_original_gldm_LargeDependenceHighGrayLevelEmphasis \| \| intra_wavelet_LLL_firstorder_10Percentile \| \| intra_original_firstorder_Kurtosis \| \| intra_wavelet_LHH_glszm_SizeZoneNonUniformity \| \| intra_wavelet_LLL_glcm_Imc2 \| \| \| --- \| --- \| --- \| --- \| --- \| --- \| --- \| --- \| |
| 3D-P | 7 | \| \| peri_wavelet_HLH_firstorder_Kurtosis \| \| --- \| \| peri_log_sigma_2_0_mm_3D_firstorder_Kurtosis \| \| peri_wavelet_LHL_firstorder_Maximum \| \| peri_wavelet_LLH_glszm_LargeAreaLowGrayLevelEmphasis \| \| peri_wavelet_LLH_glcm_Imc2 \| \| peri_wavelet_LLL_firstorder_Median \| \| peri_original_shape_SurfaceVolumeRatio \| \| \| --- \| --- \| --- \| --- \| --- \| --- \| --- \| --- \| |
| 3D-C | 11 | \| \| peri_wavelet_HLH_firstorder_Kurtosis \| \| --- \| \| peri_log_sigma_2_0_mm_3D_firstorder_Kurtosis \| \| peri_wavelet_LLH_glszm_LargeAreaLowGrayLevelEmphasis \| \| intra_wavelet_LLL_glcm_Imc2 \| \| intra_wavelet_LHH_glcm_ClusterShade \| \| intra_wavelet_LLL_firstorder_10Percentile \| \| peri_wavelet_LHL_firstorder_Maximum \| \| peri_wavelet_LLH_glcm_Imc2 \| \| intra_log_sigma_2_0_mm_3D_glcm_ClusterShade \| \| intra_original_gldm_LargeDependenceHighGrayLevelEmphasis \| \| peri_original_shape_SurfaceVolumeRatio \| \| \| --- \| --- \| --- \| --- \| --- \| --- \| --- \| --- \| --- \| --- \| --- \| --- \| |

| Table S5 Results of Delong's Test for Models’ Training Performance across LVSI | | | | | | |
| --- | --- | --- | --- | --- | --- | --- |
| Model | 2D-I | 2D-P | 2D-C | 3D-I | 3D-P | 3D-C |
| 2D-I | 1 | 0.2529 | 0.0061 | 0.0014 | 0.9454 | 0.8646 |
| 2D-P | 0.2529 | 1 | 0.0009 | 0.1113 | 0.2491 | 0.2491 |
| 2D-C | 0.0061 | 0.0009 | 1 | 0.0001 | 0.0969 | 0.1259 |
| 3D-I | 0.0014 | 0.1113 | 0.0001 | 1 | 0.0001 | 0.0001 |
| 3D-P | 0.9454 | 0.2491 | 0.0969 | 0.0001 | 1 | 0.7190 |
| 3D-C | 0.8646 | 0.2329 | 0.1259 | 0.0001 | 0.7190 | 1 |

| Table S6 Results of Delong's Test for Models’ Validation Performance across LVSI | | | | | | |
| --- | --- | --- | --- | --- | --- | --- |
| Model | 2D-I | 2D-P | 2D-C | 3D-I | 3D-P | 3D-C |
| 2D-I | 1 | 0.9384 | 0.8388 | 0.9665 | 0.0746 | 0.0670 |
| 2D-P | 0.9384 | 1 | 0.7669 | 0.9733 | 0.1155 | 0.1122 |
| 2D-C | 0.8388 | 0.7669 | 1 | 0.8504 | 0.1216 | 0.1086 |
| 3D-I | 0.9665 | 0.9733 | 0.8504 | 1 | 0.0494 | 0.0177 |
| 3D-P | 0.0746 | 0.1155 | 0.1216 | 0.0494 | 1 | 0.7182 |
| 3D-C | 0.0670 | 0.1122 | 0.1086 | 0.0177 | 0.7182 | - |

| Table S7 Results of Delong's Test for Models’ Training Performance across DMI | | | | | | |
| --- | --- | --- | --- | --- | --- | --- |
| Model | 2D-I | 2D-P | 2D-C | 3D-I | 3D-P | 3D-C |
| 2D-I | 1 | 0.0001 | 0.0001 | 0.1610 | 0.0219 | 0.0011 |
| 2D-P | 0.0001 | 1 | 0.0001 | 0.0001 | 0.0001 | 0.0001 |
| 2D-C | 0.0001 | 0.0001 | 1 | 0.0001 | 0.8305 | 0.6898 |
| 3D-I | 0.1610 | 0.0001 | 0.0001 | 1 | 0.0001 | 0.0001 |
| 3D-P | 0.0219 | 0.0001 | 0.8305 | 0.0001 | 1 | 0.1569 |
| 3D-C | 0.0011 | 0.0001 | 0.6898 | 0.0001 | 0.1569 | 1 |

| Table S8 Results of Delong's Test for Models’ Validation Performance across DMI | | | | | | |
| --- | --- | --- | --- | --- | --- | --- |
| Model | 2D-I | 2D-P | 2D-C | 3D-I | 3D-P | 3D-C |
| 2D-I | 1 | 0.5932 | 0.0450 | 0.5705 | 0.0310 | 0.1628 |
| 2D-P | 0.5932 | 1 | 0.3014 | 0.3033 | 0.5806 | 0.4658 |
| 2D-C | 0.0450 | 0.3014 | 1 | 0.0310 | 0.7991 | 0.9242 |
| 3D-I | 0.5705 | 0.3033 | 0.0310 | 1 | 0.0191 | 0.0026 |
| 3D-P | 0.2574 | 0.5806 | 0.7991 | 0.0191 | 1 | 0.6468 |
| 3D-C | 0.1628 | 0.4658 | 0.9242 | 0.0026 | 0.6468 | 1 |

| Table S9 Results of Delong's Test for Models’ Training Performance across FIGO Stage | | | | | | |
| --- | --- | --- | --- | --- | --- | --- |
| Model | 2D-I | 2D-P | 2D-C | 3D-I | 3D-P | 3D-C |
| 2D-I | 1 | 0.0049 | 0.6818 | 0.5513 | 0.0306 | 0.0001 |
| 2D-P | 0.0049 | 1 | 0.0131 | 0.2657 | 0.0002 | 0.0001 |
| 2D-C | 0.6818 | 0.0131 | 1 | 0.1230 | 0.5005 | 0.0061 |
| 3D-I | 0.5513 | 0.2657 | 0.1230 | 1 | 0.0496 | 0.0001 |
| 3D-P | 0.0306 | 0.0002 | 0.5005 | 0.0496 | 1 | 0.0009 |
| 3D-C | 0.0001 | 0.0001 | 0.0061 | 0.0001 | 0.0009 | 1 |

| Table S10 Results of Delong's Test for Models’ Validation Performance across FIGO Stage | | | | | | |
| --- | --- | --- | --- | --- | --- | --- |
| Model | 2D-I | 2D-P | 2D-C | 3D-I | 3D-P | 3D-C |
| 2D-I | 1 | 0.9407 | 0.0450 | 0.5705 | 0.2874 | 0.0379 |
| 2D-P | 0.9407 | 1 | 0.7492 | 0.5685 | 0.3125 | 0.0578 |
| 2D-C | 0.5668 | 0.7492 | 1 | 0.3833 | 0.1975 | 0.0234 |
| 3D-I | 0.5513 | 0.5685 | 0.3833 | 1 | 0.1975 | 0.0234 |
| 3D-P | 0.2874 | 0.3125 | 0.1975 | 0.5604 | 1 | 0.0744 |
| 3D-C | 0.0379 | 0.0578 | 0.0234 | 0.0390 | 0.0744 | 1 |
